# Bilateral posterior Quadratus Lumborum block for pain relief after cesarean delivery: a randomized controlled trial

**DOI:** 10.1186/s12871-021-01309-6

**Published:** 2021-03-25

**Authors:** Pawinee Pangthipampai, Sukanya Dejarkom, Suppachai Poolsuppasit, Choopong Luansritisakul, Suwida Tangchittam

**Affiliations:** 1 Department of Anesthesiology, Faculty of Medicine Siriraj Hospital, Mahidol University, 2 Wanglang Road, Bangkoknoi, Bangkok, 10700 Thailand; 2 Department of Anesthesiology, Police General Hospital, Bangkok, Thailand

**Keywords:** Bilateral posterior quadratus lumborum block, Ultrasound-guided QLB, Pain relief, Cesarean delivery

## Abstract

**Background:**

Achieving optimal analgesia with few side effects is the goal of pain management after cesarean delivery. Intrathecal (IT) morphine is the current standard but ultrasound-guided quadratus lumborum block (QLB) may offer superior pain control with fewer side effects. This study compared the pain-free period after cesarean delivery among parturients who received spinal block with IT morphine, with IT morphine and bilateral QLB, or only bilateral QLB.

**Methods:**

Parturients having elective cesarean delivery under spinal block were randomized and allocated into IT morphine 0.2 mg with sham QLB (Group IT), IT morphine 0.2 mg and bilateral QLB with 0.25% bupivacaine 25 ml in each side (Group IT+QLB), or bilateral QLB with 0.25% bupivacaine 25 ml in each side (Group QLB). A PCA pump was connected after completion of the QLB or sham block. The first time to PCA morphine requirement was recorded and compared.

**Results:**

Eighty parturients were included. Analysis of Group QLB was terminated early because at the second interim analysis, median pain-free period was significantly shorter in Group QLB [hours (95%CI): 2.50 (1.04–3.96) in Group IT vs. 7.75 (5.67–9.83) in IT+QLB vs. 1.75 (0.75–2.75) in QLB (*p* < 0.001)]. The median (min, max) amount of morphine required during 24 h was 5.5 (0–25) in Group IT vs. 5.0 (0–36) in IT+QLB vs. 17.5 (1–40) mg in Group QLB (*p* < 0.001). In the final analysis the median pain-free period was 2.50 (1.23–3.77) hours (95%CI) in Group IT (*n* = 27) vs. 8.02 (5.96–10.07) in IT+QLB (*n* = 28). (*p* = 0.027).

**Conclusion:**

US-QLB used in conjunction with IT morphine yielded a statistically significant longer median pain-free period compared with standard IT morphine alone. However, QLB alone provided inferior pain control compared with standard IT morphine. When combined with IT morphine, QLB could provide additional analgesic benefit as a part of multimodal analgesic regimen, especially during the early postoperative period.

**Trial registration:**

ClinicalTrials.gov no. NCT03199170 Date registered on June 22, 2017. Prospectively registered.

## Background

Achieving optimal analgesia with the fewest possible side effects is the goal of postoperative pain management after cesarean delivery. Effective pain relief improves maternal ambulation and reduces the risk of thromboembolism, accelerates and facilitates breastfeeding, improves mother-child interaction, and decreases the risk of chronic pain and depression [1–4]. Intrathecal (IT) morphine is the standard method for postoperative pain control following spinal anesthesia for cesarean delivery [3, 5] but increases the risk of maternal pruritus, nausea and vomiting and rare devastating respiratory depression [1, 6, 7].

As a result of recent advances in ultrasound (US)-guided regional anesthesia, the popularity of abdominal wall block has dramatically increased during the last decade. US-guided transversus abdominis plane block (TAPB) has been shown to be an effective component of multimodal analgesia in parturients who are unable to receive neuraxial opioids or whose pain is not adequately controlled. However, there is no significant analgesic or opioid-sparing benefit of routine TAPB after cesarean delivery in patients who receive intrathecal morphine [8].

US-guided quadratus lumborum block (QLB) reflects the continued evolution of US-guided TAPB. The upward spreading of local anesthetic into the thoracic paravertebral space [9–13] to mechanoreceptors and the network of sympathetic fibers within the thoracolumbar fascia [14], or the spread of local anesthetic via the splanchnic nerves to the celiac ganglion or sympathetic chain [15] have been proposed as possible mechanisms for the more extensive abdominal analgesia compared to US-guided TAPB. To date, very few studies have compared the efficacy of US-guided QLB with that of IT morphine. One study found that QLB at the lumbar interfascial triangle had a longer duration of time to first morphine dose than 0.1 mg of IT morphine [16]. A different study reported that IT morphine resulted in better postoperative analgesia than posterior QLB. Specifically, the addition of posterior QLB to a multimodal analgesic regimen including 0.1 mg IT morphine was associated with similar severity of postoperative pain [17].

Accordingly, we aimed to evaluate whether US-guided posterior QLB (QLB type 2) at the lumbar interfascial triangle [14, 18] could provide additional benefits to the current analgesic regimen after cesarean delivery. The primary objective was to compare the pain-free period after cesarean delivery among parturients who received spinal block with IT morphine 0.2 mg, with IT morphine and bilateral QLB, or with only bilateral QLB. The pain free-period defines as time to first morphine requirement via a patient-controlled analgesia (PCA) pump. The secondary outcomes were cumulative morphine consumption within 48 h and side effects between groups.

## Methods

This study was conducted during March 2017 to October 2018. The Siriraj Institutional Review Board (SiRB) of the Faculty of Medicine Siriraj Hospital, Mahidol University, Bangkok, Thailand approved the study (COA no. 817/2559[EC1]). The study report has been prepared in accordance with the Consolidated Standards of Reporting Trials (CONSORT) guidelines. Prior written informed consent was obtained. Parturients were eligible if they were having an elective cesarean delivery with a low transverse incision under spinal block. Inclusion criteria were American Society of Anesthesiologists physical status I or II and a normal singleton pregnancy with a gestation of at least 37 weeks. Patients with a history of chronic pain, allergy to the study drugs (local anesthetics, morphine, paracetamol, and/or ibuprofen), local infection at one or both flank areas (the puncture sites for QLB), requiring additional analgesic drugs and/or general anesthesia to complete operation or having an inability to comprehend or use the numerical rating scale (NRS) for pain assessment and/or the patient-controlled analgesia (PCA) pump were excluded. This trial was registered with ClinicalTrials.gov (reg. no. NCT03199170).

A computer-generated block of six randomization scheme was used to allocate parturients into each of three groups: IT morphine 0.2 mg with sham QLB (Group IT), IT morphine 0.2 mg and bilateral QLB with 0.25% bupivacaine 25 ml and adrenaline 1:250,000 in each side (Group IT+QLB), or bilateral QLB with 0.25% bupivacaine 25 ml and adrenaline 1:250,000 in each side (Group QLB). Randomization assignments were placed in envelopes and sealed. On the day of the operation, the sealed opaque envelope containing that patient’s group allocation was opened before the patient was taken into the operating theater. Surgeons, patients, and the research nurse who evaluated patients postoperatively were all blinded to the group assignment. The anesthesiologist caring for the woman and the anesthesiologist performing the QLB were not blinded.

All patients were instructed how to use the Numeric Rating Scale (NRS) (NRS: 0, no pain to 10, worst imaginable pain) for pain assessment during the preoperative visit. Pain with movement was assessed during ambulation. Patients received 150 mg ranitidine by mouth in the evening before surgery, and again in the morning of surgery. With the patient in the lateral decubitus or sitting position, spinal block was performed at the levels of L3–4 and L4–5 intervertebral spaces using 0.5% hyperbaric bupivacaine 2–2.2 ml depending on the judgment of the anesthesiologist responsible for that patient. After the baby was delivered, ondansetron 8 mg was given intravenously. Before ultrasound scanning and performing QLB blocks, pinprick sensation test was used to check the patient’s pain level. All QLB and sham blocks were performed in the postoperative care unit immediately after cesarean delivery, before the patients experienced any postoperative pain or pain during the QLB procedure. One anesthesiologist (PP) with more than 5 years of experience in performing US-guided regional anesthesia who not involve with the data collection performed all blocks. Postoperatively, all parturients received regular acetaminophen (1 g orally every 6 h) and ibuprofen (400 mg orally every 8 h). For breakthrough pain, intravenous morphine via a patient-controlled analgesia (PCA) pump was used with the setting of bolus morphine 1 mg, a lockout of 5 min, and a 4-h-maximum dose of 30 mg.

### Quadratus lumborum block (QLB) administration

A FUJIFILM SonoSite Edge ultrasound unit (FUJIFILM SonoSite, Inc., Bothell, WA, USA) with a 2–5 MHz curved transducer was used to identify all relevant muscles and fascial layers. Patients were positioned in the supine position, and both iliac crests were slightly elevated by pillows placed underneath the patient’s hips. The US transducer was placed in the transverse plane on the flank of the patient cranially to the iliac crest at the level of the L3 or L4 transverse process. The muscle layers of the abdominal wall were identified. The transducer was then moved posteriorly to visualize the aponeurosis of the transversus abdominis muscle. The pararenal fat and the quadratus lumborum muscle were imaged medial to the aponeurosis. A 20-gauge 80 mm Stimuplex® Ultra 360 needle (B. Braun Melsungen AG, Hessen, Germany) was advanced in-plane under US guidance in an anteroposterior direction through the muscle layers of the abdominal wall. The needle tip was advanced and aimed to the lumbar interfascial triangle on the posterolateral aspect of the quadratus lumborum muscle as described by Blanco R [14]. One to 2-ml test dose of normal saline was injected to confirm appropriate positioning. If necessary, the needle was then repositioned. On each side, 25 ml of 0.25% bupivacaine with adrenaline 1:250,000 was then injected with aspiration repeated after every 5 ml of medication injected. A sham block using subcutaneous injection of 0.5 ml sterile normal saline injection was performed in group IT at the same area using ultrasound transducer pressure that was intended to simulate a real block procedure.

A PCA pump was connected to each parturient after completion of the QLB or sham block for 48 h (study period). All parturients were instructed to press the hand-held button to activate the PCA when they experienced pain-related discomfort an NRS score of 4 out of 10. Parturients were asked to record their level of pain at 4, 6, 12, 24, and 48 h after QLB or sham block. A research nurse that was blinded to group assignment assessed and confirmed each parturient’s report the next day. The puncture sites were also examined, and the patient was assessed for block-related complications. The time to first PCA use, daily PCA demand, delivery counts, and cumulative dose were extracted from the internal memory of the pump.

The severity and management of all complications were recorded and analyzed. Sedation was rated as 0 (none), 1 (mild, occasionally drowsy, easy to arouse), 2 (moderate, constantly or frequently drowsy, easy to arouse), 3 (severe, somnolent, difficult to arouse), or S (sleeping, easy to arouse). Respiratory depression was defined as a respiratory rate lower than 8 breaths/min, and was rated as absent or present. Nausea, vomiting, and pruritus were rated as 0 (none), 1 (mild, requiring no treatment), 2 (moderate with resolution via medication), or 3 (severe and persistent despite medication). Muscle weakness of the lower extremities and sign of local anesthetic systemic toxicity, both of which have been reported as side effects from QLB in previous reports, were also asked and examined. Straight leg raising test to evaluate quadriceps was used.

Since QLB is a relatively new technique for post-cesarean analgesia at our center, interim analysis was performed after 10 and 20 women were recruited into each study arm. The aim is to compare analgesia with the current standard analgesic technique and to ensure patient safety. The statistician involved in the analysis remained blinded to the group allocation until the final analysis was complete.

### Sample size calculation

The primary hypothesis was that the pain-free period would be longer when QLB was combined with IT morphine compared to standard IT morphine alone. The reference data we used to calculate our sample size was reported by Triyasunant, et al. That study found the median pain-free period (median survival time) after cesarean delivery under IT morphine 0.2 mg alone to be 2 h (120 min) [19]. We considered an increase in the pain-free duration (from 2 h to 6 h [360 min]) to be a clinically significant improvement. Power analysis was performed to detect a clinically significant increase of 150% in the pain-free period with a power of 80%, alpha of 0.05 and 95% significant level. The sample size calculation was performed based on previously reported median survival time and interim analyses (3 looks of equal sample size). The calculated sample size was 24 patients for each of the 3 groups. To compensate for missing data or dropout for any cause, 30 patients per group were enrolled.

### Statistical analysis

Continuous data are reported as mean ± standard deviation for normally distributed data, and as median and interquartile range for non-normally distributed data. Categorical data are reported as frequency and percentage. Comparisons between groups were performed using the independent t-test, Chi-square test, Mann-Whitney U test, one-way analysis of variance (ANOVA), and the Kruskal-Wallis test. A Kaplan-Meier curve for time to first PCA was constructed and tested among the three groups using log-rank test to give equal weight to all differences. At the final analysis comparing two Kaplan-Meier curves notable differences in the early time point were apparent and therefore results from the Gehan-Breslow and the Tarone-Ware tests using different weights were also presented. Gehan-Breslow test was chosen because it gives more weight to earlier failures (require PCA morphine), while log-rank test gives equal weight to all failures and Tarone-Ware test falls in between. A *p*-value of less than 0.05 was considered statistically significant. Statistical analyses were performed using SPSS Statistics version 18 (SPSS, Inc., Chicago, IL, USA). The interim analyses were performed after 10 and 20 women were recruited into each arm by a blinded statistician using Kaplan-Meier curve to evaluate the pain-free period, and using one-way ANOVA to compare the amount of morphine consumption during 24-h period after cesarean delivery.

## Results

**Fig. 1:**
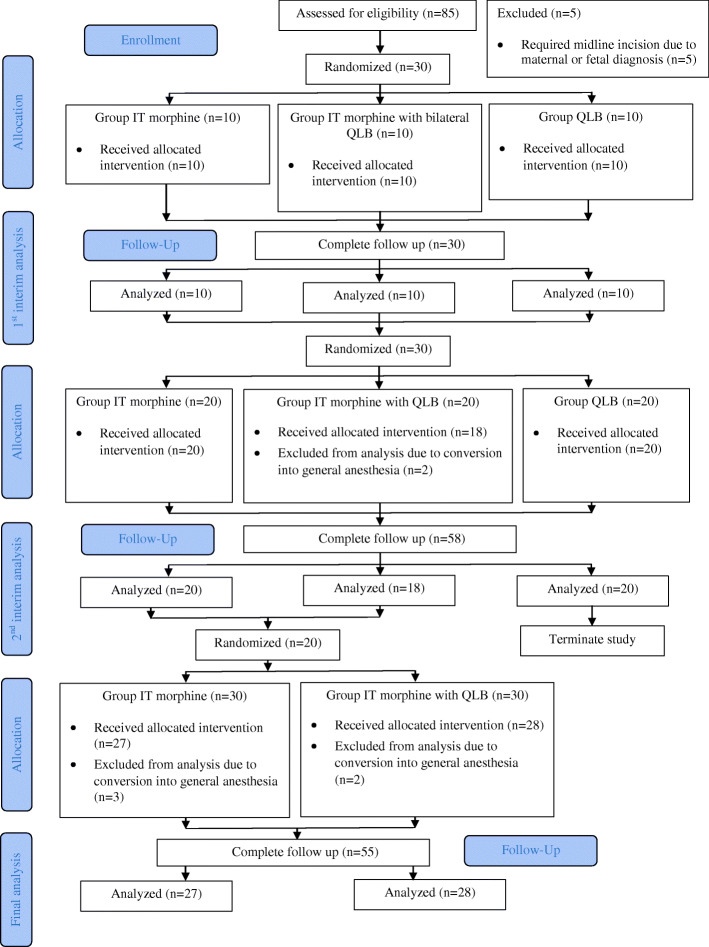
Consolidated Standards of Reporting Trials (CONSORT) flow diagram

Eighty-five parturients were invited to participate in this study. Five parturients were excluded because of planning for midline incision due to their diagnosis. The remaining 80 parturients were randomized into three arms using block of six randomization until the second interim analysis, at which point the QLB group was terminated. The interim analyses were performed after 10 and 20 women were recruited to each arm. Analysis of Group QLB was terminated early because Kaplan-Meier survival analysis showed the elapsed time between completion of the block and the first administration of morphine by PCA (pain-free period) to be significantly shorter in Group QLB at the second interim analysis. A CONSORT flow diagram describing the study protocol is shown in Fig. 1. At the postoperative care unit immediately after cesarean delivery, no local anesthetic skin infiltration for QLB placement was required in any of the parturients.

### Interim analysis

The first interim analysis revealed the pain-free period to be 2.50 (1.34–3.66) [hours (95%CI)] in Group IT vs. 7.75 (5.68–9.82) in IT+QLB vs. 1.75 (0.33–3.17) in QLB (overall *p* = 0.002). Log-rank test for pairwise analysis revealed the differences in Group IT vs. IT+QLB (*p* = 0.318), IT vs. QLB (*p* = 0.166), and IT+QLB vs. QLB (*p* < 0.001). The mean amount of morphine required during 24 h was 10.70 ± 9.04 mg in Group IT vs. 7.40 ± 10.35 IT+QLB vs. 17.00 ± 5.94 QLB (*p* = 0.057).

**Fig. 2:**
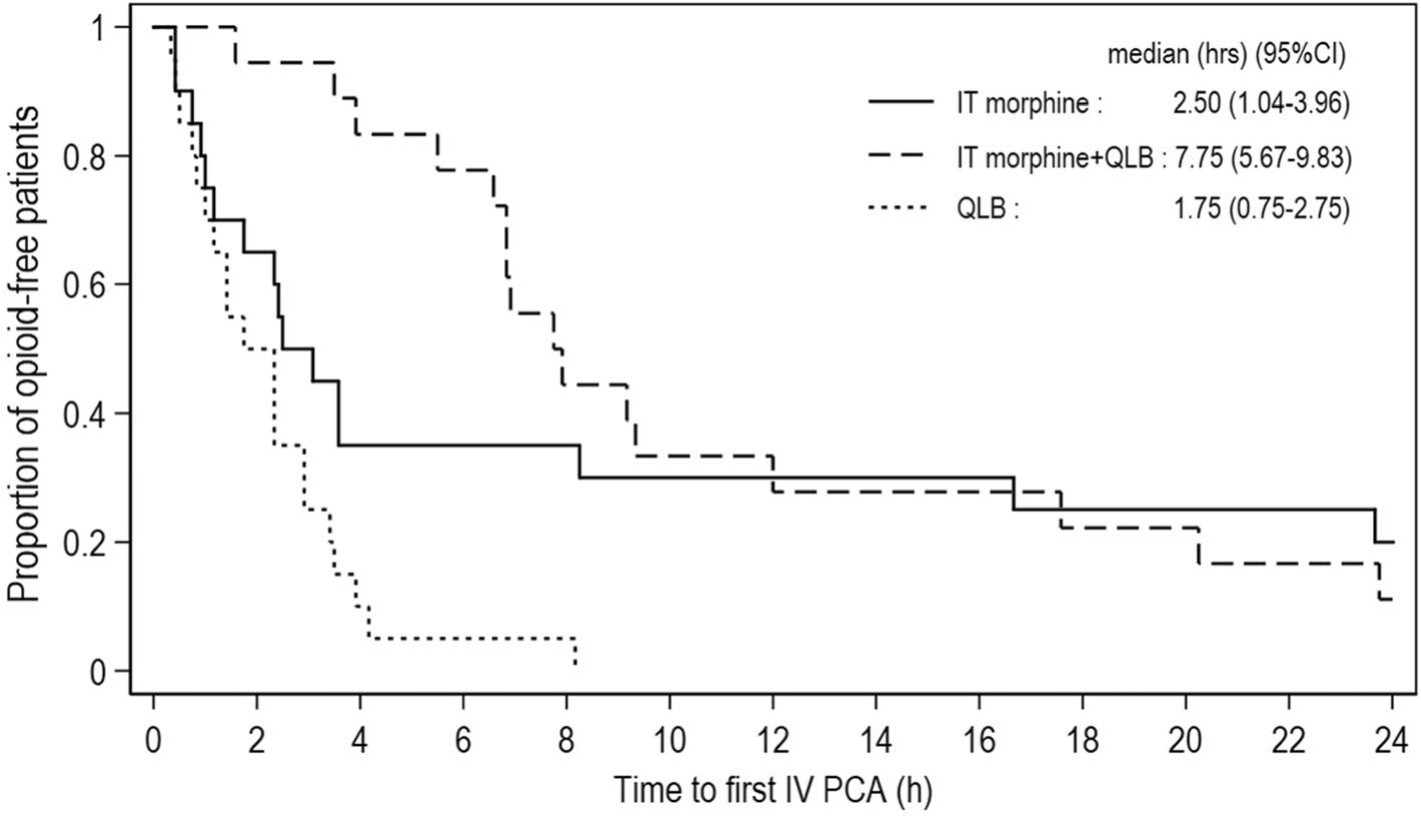
Kaplan-Meier plot of time to first request for morphine (pain-free period) at the 2nd interim analysis, Log-rank overall *p* < 0.001. Abbreviations: IT, intrathecal; QLB, quadratus lumborum block; IV, intravenous; PCA, patient-controlled analgesia; h, hours

**Fig. 3:**
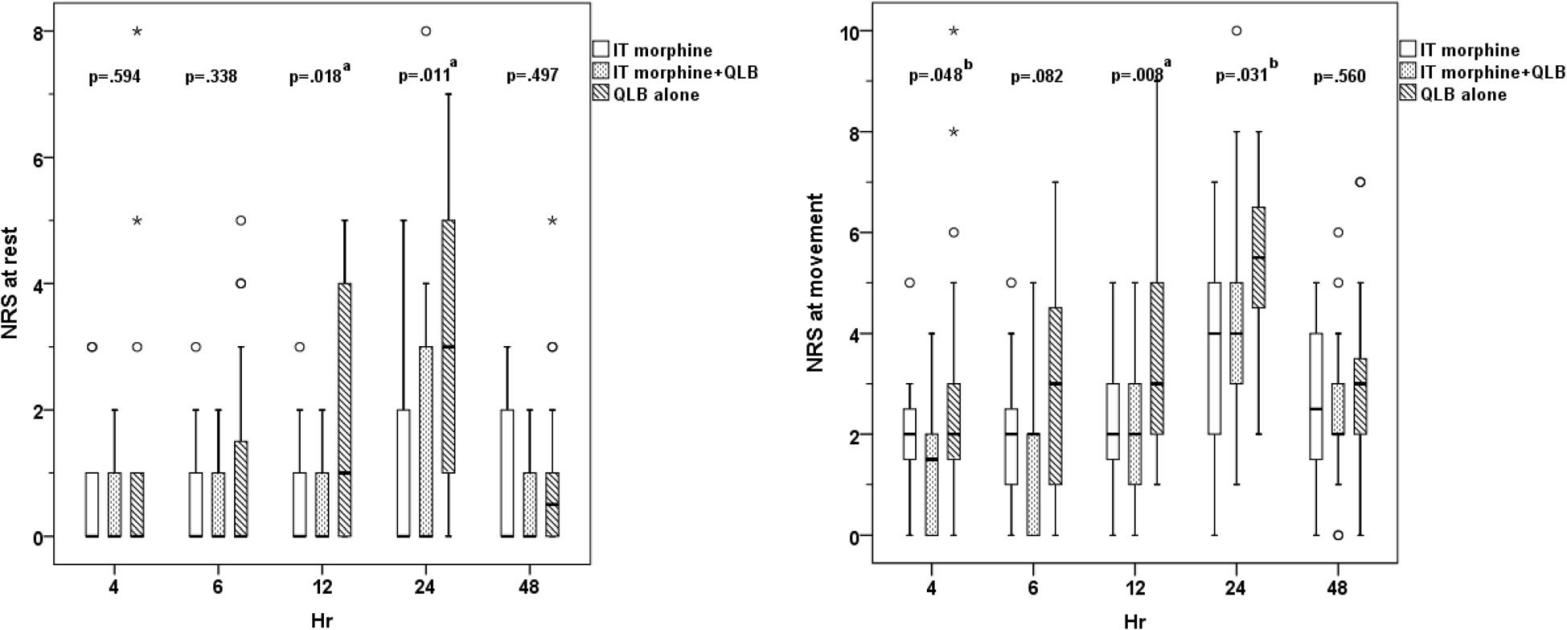
Box plot of pain scores both at rest and at movement overtime after cesarean delivery between Group IT, IT+QLB and QLB at the 2nd interim analysis. Abbreviations: IT, intrathecal; QLB, quadratus lumborum block; NRS, Numeric Rating Scale*. Kruskal-Wallis with Dunn’s* post hoc *test with significances indicated by: a, both Group IT and IT + QLB* vs. *QLB, p < 0.05, b, IT* vs. *QLB, p < 0.05*

Demographic and clinical data at the second interim analysis (after 20 parturients were recruited into each of the 3 arms) are shown in Table 1. Two parturients in Group IT+QLB were excluded from analysis due to conversion to general anesthesia. There were no patients that required additional intraoperative analgesia. The Kaplan-Meier survival curves showing the pain-free periods in all groups are shown in Fig. 2. The median pain-free period was 2.50 (1.04–3.96) [hours (95% CI)] in Group IT vs. 7.75 (5.67–9.83) IT+QLB vs. 1.75 (0.75–2.75) QLB (overall *p* < 0.001). Log-rank test for pairwise analysis revealed the differences in IT vs. IT+QLB (*p* = 0.486), IT vs. QLB (*p* = 0.019), and IT+QLB vs. QLB (*p* < 0.001). The NRS pain scores both at rest and at movement 4, 6, 12, 24, and 48 h postoperatively between groups were shown in Fig. 3. The median (min, max) amount of morphine required during 24 h was 5.5 (0–25) vs. 5.0 (0–36) vs. 16.5 (1–44) mg in IT vs. IT+QLB vs. QLB, respectively (*p* = 0.001). Cumulative morphine use (mg) and demands between groups at 2nd interim analysis are shown in Table 2.

**Table 1:** Demographic and clinical data at the 2nd interim analysis

Data	IT morphine (***n*** = 20)	IT morphine with QLB (***n*** = 18)	QLB (n = 20)	***p***-value
Age (yr)	32.25 ± 5.26	31.06 ± 6.58	32.70 ± 6.70	0.705
Body mass index (kg/m^2^)	27.90 ± 2.98	28.92 ± 4.05	28.17 ± 4.15	0.694
Operation				0.806
C/S	16 (80.0%)	13 (72.2%)	16 (80.0%)	
C/S with TS	4 (20.0%)	5 (27.8%)	4 (20.0%)	
Operative time (min)	57.75 ± 16.18	60.28 ± 17.94	68.50 ± 15.82	0.113
Total morphine in 24 h (mg)	5.5 (0–25)	5.0 (0–36)	17.5 (1–40)	< 0.001
Pruritus				0.007
0	9 (45.0%)	15 (83.3%)	20 (100%)	
1	8 (40.0%)	2 (11.1%)	0 (0.0%)	
2	2 (10.0%)	1 (5.6%)	0 (0.0%)	
3	1 (5.0%)	0 (0.0%)	0 (0.0%)	
PONV				0.380
0	19 (95.0%)	18 (100%)	20 (100%)	
1	1 (5.0%)	0 (0.0%)	0 (0.0%)	

Data presented as mean ± standard deviation, number and percentage, or median and range (min, max). A *p*-value< 0.05 indicates statistical significance.

**Abbreviations:** *IT* intrathecal, *QLB* quadratus lumborum block, *C/S* cesarean section, *TS* tubal sterilization, *PONV* postoperative nausea and vomiting

*Group QLB had significantly higher morphine consumption in 24 h than both IT (p = 0.003) and IT + QLB (p = 0.002). There was no significant difference in morphine consumption between IT and IT + QLB (p = 1.000).*

*Group IT had a significantly higher number of patients with pruritus than both IT + QLB (p = 0.020) and QLB (p < 0.001). There was no significant difference in pruritus between IT + QLB and QLB (p = 0.480).*

**Table 2:** Cumulative morphine use (mg) and cumulative morphine demands at the 2nd interim analysis

	IT morphine (***n*** = 20)	IT morphine with QLB (***n*** = 18)	QLB (***n =*** 20)	***p***-value
PCA morphine delivery (1 dose = 1 mg)				
24 h	5.5 (0–25)	5 (0–36)	16.5 (1–44)	0.001^a^
48 h	5.5 (0–37)	6 (0–40)	20 (1–46)	0.006^b^
PCA morphine demand				
24 h	5.5 (0–38)	5 (0–37)	19 (2–65)	< 0.001^a^
48 h	5.5 (0–42)	6 (0–39)	22.5 (2–67)	0.003^a^

Data presented as median and range (min, max). Kruskal-Wallis with Dunn’s post hoc test with significances indicated by: a, both Group IT and IT+QLB vs. QLB, *p* < 0.05, b, IT vs. QLB, *p* < 0.05.

**Abbreviations:** *IT* intrathecal, *QLB* quadratus lumborum block, *PCA* patient-controlled analgesia

### Final analysis

**Fig. 4:**
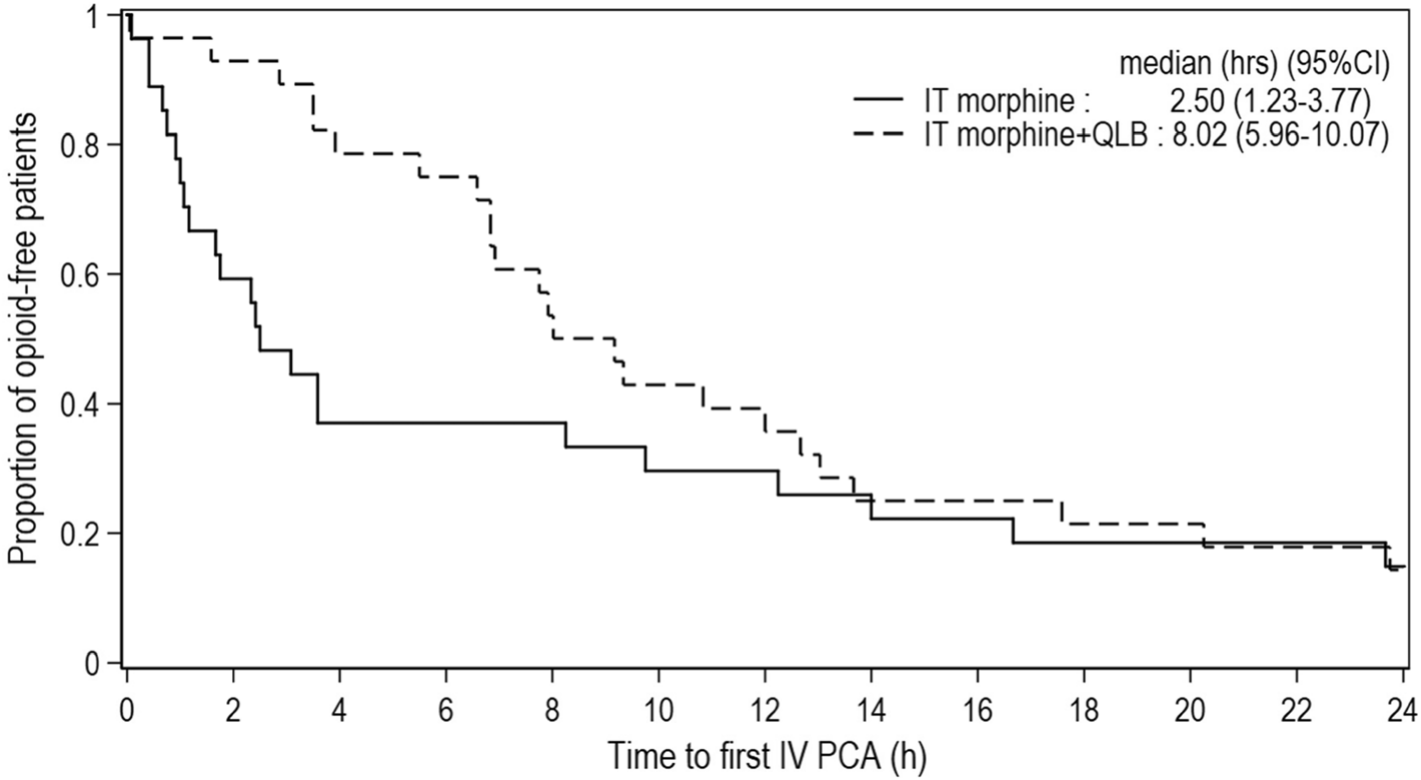
Kaplan-Meier plot of time to first request for morphine (pain-free period) at the final analysis, Log-rank test: *p* = 0.238. Abbreviations: IT, intrathecal; QLB, quadratus lumborum block; IV, intravenous; PCA, patient-controlled analgesia; h, hours

**Fig. 5:**
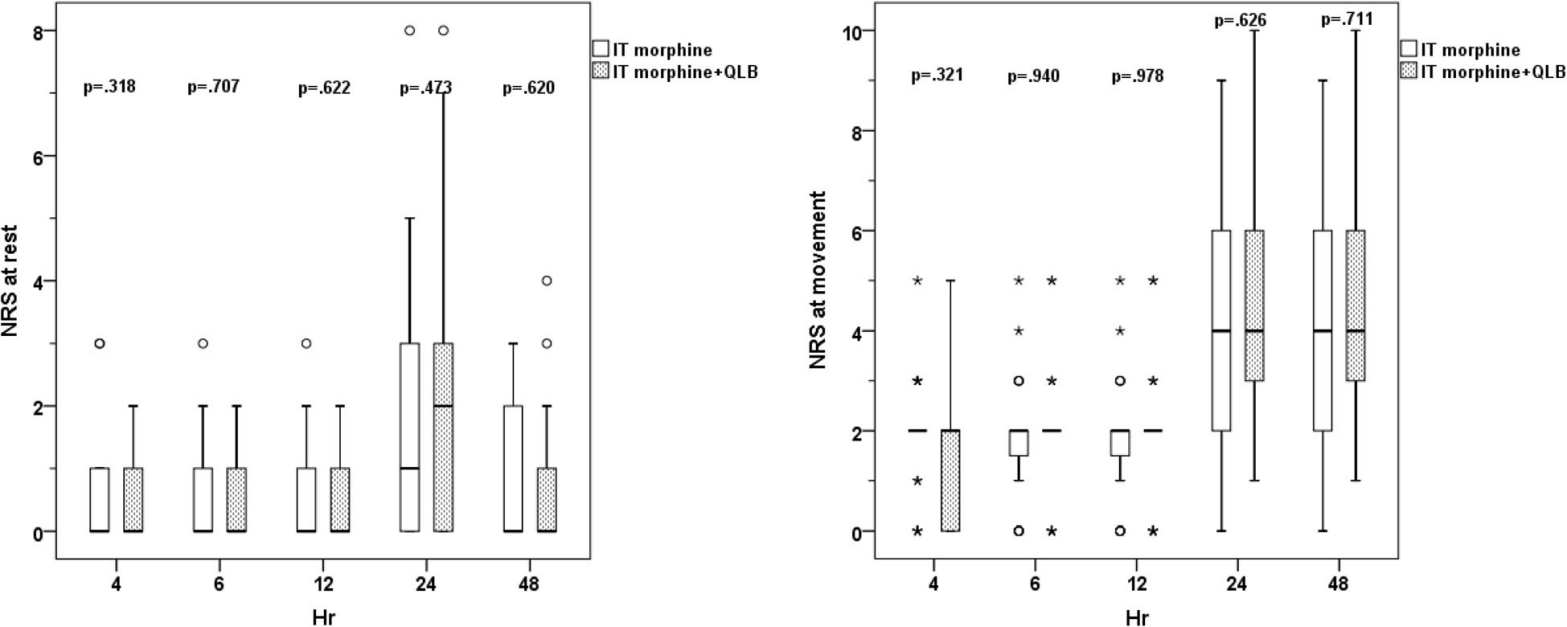
Box plot of pain scores both at rest and at movement overtime after cesarean delivery between Group IT and IT+QLB at the final analysis. Abbreviations: IT, intrathecal; QLB, quadratus lumborum block; NRS, Numeric Rating Scale. *Mann-Whitney U test were used and a p-value < 0.05 indicates statistical significance*

After Group QLB was terminated, Group IT and IT+QLB were continued in order to analyze whether QLB could be effective for improving postoperative analgesia by extending the pain-free period. Randomization was resumed until there were 30 patients allocated to each of the two remaining study groups. Three patients in Group IT and two patients in IT+QLB were excluded from the analysis due to conversion to general anesthesia. Demographic, surgical, morphine requirement, and side effect data are shown in Table 3. The Kaplan-Meier survival curves of pain-free periods revealed a large difference in the first 6 h then became smaller afterwards. (Fig. 4) Therefore, more weight was assigned to the early difference using the Gehan-Breslow and Tarone-Ware tests rather than the conventional log-rank test. The median pain-free period or median time to first request for IV-PCA morphine was 2.50 (1.23–3.77) [hours (95% CI)] in Group IT, and 8.02 (5.96–10.07) in IT+QLB (Gehan-Breslow *p* = 0.027 vs. Tarone-Ware *p* = 0.076 vs. log-rank *p* = 0.238). The NRS at 4, 6, 12, 24, and 48 h between groups are shown in Fig. 5. The proportion of patients without morphine requirement, cumulative morphine use (mg) and demands between groups are shown in Table 4.

**Table 3:** Demographic and clinical data at the final analysis

Data	IT morphine (***n*** = 27)	IT morphine with QLB (***n =*** 28)	***p***-value
Age (yr)	31.89 ± 4.93	30.68 ± 6.11	0.424
Body mass index (kg/m^2^)	28.76 ± 3.51	28.22 ± 3.97	0.593
Operation			0.937
C/S	20 (74.1%)	21 (75.0%)	
C/S with TS	7 (25.9%)	7 (25.0%)	
Operative time (min)	63.89 ± 18.26	64.29 ± 18.65	0.937
Total morphine in 24 h (mg)	5 (0–56)	5 (0–36)	0.565
Pruritus			0.605
0	15 (55.6%)	19 (67.9%)	
1	9 (33.3%)	8 (28.6%)	
2	2 (7.4%)	1 (3.6%)	
3	1 (3.7%)	0 (0.0%)	
PONV			0.491
0	26 (96.3%)	28 (100%)	
1	1 (3.7%)	0 (0.0%)	

Data presented as mean ± standard deviation, number and percentage, or median and range (min, max). A *p*-value< 0.05 indicates statistical significance.

**Abbreviations:** *IT* intrathecal, *QLB* quadratus lumborum block, *C/S* cesarean section, *TS* tubal sterilization, *PONV* postoperative nausea and vomiting

**Table 4:** The proportion of patients without morphine requirement, cumulative morphine use (mg) and cumulative morphine demands at the final analysis

	IT morphine (***n =*** 27)	IT morphine with QLB (***n*** = 28)	***p***-value
Without morphine requirement: n (%)			
6 h	10 (37%)	21 (75%)	0.005
8 h	10 (37%)	15 (53.6%)	0.218
10 h	8 (29.6%)	12 (42.8%)	0.308
12 h	8 (29.6%)	10 (35.7%)	0.631
24 h	4 (14.8%)	4 (14.3%)	0.956
PCA morphine delivery			
24 h	5 (0–56)	4.5 (0–36)	0.548
48 h	7 (0–78)	6.5 (0–44)	0.768
PCA morphine demand			
24 h	6 (0–61)	4.5 (0–37)	0.494
48 h	8 (0–84)	6.5 (0–39)	0.933

Data presented as *n* (%), median and range (min, max). A *p*-value< 0.05 indicates statistical significance

**Abbreviations**: *IT* intrathecal, *QLB* quadratus lumborum block, *PCA* patient-controlled analgesia

No patients in Group QLB experienced pruritus compared with other two groups (55% in Group IT and 16.7% in IT+QLB) at the 2nd interim analysis (Table 1). However, the number of parturients who experienced postoperative nausea and vomiting was comparable among the study groups. All patients had a sedation score of either 0 or 1. No respiratory depression was observed in any study patient. No parturients experienced muscle weakness of the lower extremities or sign of local anesthetic systemic toxicity from QLB.

## Discussion

This study has been evaluated additional benefits of US-guided posterior QLB (QLB type 2) to the current analgesic regimen (IT morphine) after cesarean delivery by comparing the pain-free period after cesarean delivery. The results showed the improvement in the pain-free period during the early postoperative period when QLB was combined with IT morphine. However, posterior QLB alone had a significantly shorter pain-free period compared with IT morphine alone, and patients required significantly more morphine during the first 24 h.

Compared with IT morphine alone, posterior QLB alone had a significantly shorter pain-free period, and patients required significantly more morphine during the first 24 h. This result is in contrast to the findings of Salama ER, et al. [16] who found the time to first morphine dose to be significantly longer with QLB than IT morphine. Salama ER, et al. reported a median time of 8 h in IT morphine vs. 17 in QLB, whereas we observed the median pain free period to be 2.50 h in Group IT morphine vs. 1.75 in QLB. However, Tamura T, et al.^17^ reported that initial pain scores associated with non-morphine groups were significantly higher than those of IT morphine groups. Group QLB was terminated at the 2nd interim analysis when inferior analgesia results were observed. In our study, the Kaplan-Meier curve showed a median pain-free period or median time to first request for IV-PCA morphine of 2.5 h in the Group IT morphine. Similarly, a previous study at our center reported a median time to first request for IV-PCA morphine of 2.1 h when adding IT morphine 0.2 mg [19].

However, there was an improvement in the pain-free period and opioid consumption during the early postoperative period when QLB was combined with IT morphine. The median pain-free period was 2.50 (1.23–3.77) [hours (95% CI)] in Group IT morphine and 8.02 (5.96–10.07) in IT+QLB (*p* = 0.027). Seventy-five percent of parturients in Group IT+QLB had opioid-sparing effect at 6 h after cesarean delivery, which was significantly higher compared to Group IT. The proportion of parturients with opioid-sparing effect was higher until the 12th postoperative hour, but the difference did not achieve statistical significance. The proportion of patients with opioid sparing effects in our study suggests that the analgesic effect of QLB can only last 6 to 12 h, but not 24 or 48 h as previously reported [20]. This result is similar to the findings of Mieszkowski MM, et al. [21], Krohg A, et al. [22] and Tamura T, et al. [13, 17], all of whom found the benefit of QLB to be less than 24 h. In 2018, Mieszkowski MM, et al. reported the time from C-section until the first dose of morphine to be approximately 10 h in the QLB type 1 group [21]. A 2018 study by Krohg A, et al. did not identify any clinically relevant opioid-sparing effect attributable to QLB during the 24 to 48-h period [22]. Tamura T, et al. reported the duration of the sensory loss in their study did not exceed eight hours after the posterior QLB, even at the anterior axillary line [13, 17].

Different QLB approaches with respect to the ideal point of injection may result in unequal block effects. We chose posterior QLB (QLB type 2) because it is the most superficial location, the safest approach for introducing the needle, and the supine position allows uncomplicated access to the patient. Recently from cadaveric and contrast studies, it is worthwhile to note that some of these studies could not demonstrate paravertebral spreading or even transient spreading [10, 23, 24]. This conduit was believed to facilitate upward spreading of local anesthetic into the paravertebral space to provide visceral analgesia coverage. Because thoracic paravertebral spaces contain intercostal nerves, dorsal rami and sympathetic nerves. Moreover, Kumar A, et al. [15] demonstrated the distinct sparing of paravertebral space after QLB, and highlighted that previously published images of paravertebral spread never demonstrated reverse flow from the paravertebral space.

The target location of QLB in these three studies (Salama ER, et al., Tamura T, et al. and this study) was similar (the lumbar interfascial triangle) and all compared QLB with standard IT morphine. Slightly different needle tip targets may explain these contradictory findings. Contrary to traditional peripheral nerve or plexus blocks with defined neural endpoints, the exact targets of interfascial plane blocks have not been well studied [25]. Moreover, distinction of the fascial layers usually cannot be clearly defined using current ultrasound technology. It is also not yet known if there is an optimal choice of layer for local anesthetic injection and whether this choice will affect the spread of medication or affect the clinical outcome [25]. Particularly, ultrasonographic identification of tissue planes for QL may appear different in postcesarean delivery versus non-pregnant patients [7]. The promise of more extensive abdominal analgesia compared with TAPB explains the growing interest in QLB block [18]. However, a precise explanation of the exact mechanism has not been described. We suggest that the incidence of nerve injury may be lower with interfascial plane block, but the efficacy has been difficult to predict. With the benefit of ultrasound that allows us to visualize the anatomy under the skin, interfascial plane injections that rely on indirect conduits to reach final targets might not be ideal techniques for patient care. The results of cadaveric and contrast studies seem to strongly imply that local anesthetic spread varies according to the technique used and that this may impact analgesic outcomes [26]. Regarding posterior QLB (QLB type 2) at the lumbar interfascial triangle, Carline L, et al. [23] and Yang HM, et al. [24] reported no spreading to the paravertebral space, but spreading to the transversus abdominis plane block and subcutaneous tissue was observed. Tamura T, et al. found minimal spread of local anesthetic into the paravertebral space and reported their concern that the volume of solution reaching the thoracic paravertebral space was too small to exert a strong analgesic effect on visceral pain after cesarean delivery [17].

## Limitations

This study has some limitations. First, it was impossible to establish the sensory blockade of QLB after spinal block was performed. Unfortunately, it is neither practical nor ethical to perform QLB prior to delivery in order to test the level of sensation. Nonetheless, these data could still be analyzed using the intention-to-treat principle. This will reflect the actual clinical scenarios where both success and failure can happen even in experienced hands. Second, it has been very hard to inform parturients to differentiate between somatic and visceral pain. Therefore, it still cannot conclude whether QLB could provide visceral pain coverage or not. Lastly, this study included only elective cesarean delivery with a low transverse incision (Pfannenstiel incision). Thus, the results could not generalize for all type cesarean delivery such as ones with midline incision.

## Conclusion

US-guided posterior QLB (QLB type 2) at the lumbar interfascial triangle) used in conjunction with IT morphine yielded a statistically significant longer median pain-free period compared with standard IT morphine alone. However, QLB alone provided an inferior postoperative pain control after cesarean delivery compared with standard IT morphine. QLB can provide an additional analgesic benefit when combined with IT morphine especially at early postoperative period.

## Future research

Cost effectiveness studies comparing IT morphine with QLB and standard IT morphine could further inform the criteria for using QLB. Moreover, investigating the analgesic efficacy of QLB in special groups such as chronic opioid users or those who experience severe breakthrough pain may provide additional insights.

## Data Availability

The datasets used and/or analysed during the current study are available from the corresponding author on reasonable request.

## Abbreviations

IT: Intrathecal
US: Ultrasound
TAPB: Transversus abdominis plane block
QLB: Quadratus lumborum block
NRS: Numerical rating scale
